# Simultaneous Determination of 16 Phenolic Compounds in Edible Fruits from Spontaneous Species Using HPLC-DAD

**DOI:** 10.3390/molecules30153071

**Published:** 2025-07-23

**Authors:** Liliana Ciurlă, Iuliana-Maria Enache, Antoanela Patraș

**Affiliations:** “Ion Ionescu de la Brad” Iasi University of Life Sciences (IULS), 3 Mihail Sadoveanu, Alley, 700490 Iasi, Romania; liliana.ciurla@iuls.ro (L.C.); iuliana.enache@iuls.ro (I.-M.E.)

**Keywords:** phenolic compounds, wild fruits, high-performance liquid chromatography, diode array detection, method validation

## Abstract

A high-performance liquid chromatography with diode array detection (HPLC-DAD) method was optimized and validated for the simultaneous analysis of 16 phenolic compounds, namely gallic acid, protocatechuic acid, *p*-hydroxybenzoic acid, vanillic acid, caffeic acid, catechin, chlorogenic acid, vanillin, syringic acid, coumaric acid, epicatechin, ferulic acid, sinapic acid, salicylic acid, resveratrol, and quercetin, in edible fruits from spontaneous species. Following the validation protocol, the proposed analytical method met the criteria of specificity, linearity, precision, and accuracy. The validated method was then applied for the analysis of phenolic compounds in fruits of hawthorn (*Crataegus monogyna*), cornelian cherry (*Cornus mas*), rosehip (*Rosa canina*), and blueberry (*Vaccinium myrtillus*). Of the phenolic compounds investigated, ten were identified in blueberry fruit (*Vaccinium myrtillus*), ten in cornelian cherry fruit (*Cornus mas*), nine in hawthorn fruit (*Crataegus monogyna*), and seven in rosehip fruit (*Rosa canina*). In the case of hawthorn (*Crataegus monogyna*), cornelian cherry (*Cornus mas*), and blueberry (*Vaccinium myrtillus*) fruit, epicatechin was identified as the main phenolic compound, while in rosehip (*Rosa canina*) fruit, catechin was the phenolic with the highest content.

## 1. Introduction

As secondary metabolites of plants, phenolic compounds are found throughout the plant kingdom [1] and are the second most prevalent class of organic molecules, behind cellulose [2]. Phenolic compounds exhibit varying degrees of distribution, with some being prevalent across a wide range, while others are limited to specific plant families, organs, or developmental stages [3]. Plants synthesize phenolic compounds through the phenylpropanoid metabolism by converting the precursors obtained via the shikimic acid and malonic acid pathways [2,4]. The fundamental structural unit of phenolic compounds is represented by an aromatic ring functionalized with one or more hydroxyl groups [2,5,6,7]. There are over 8000 known structures of phenolic compounds [2,5] that range from simple molecules as phenolic acids, through flavonoids that consist of several groups, to complex polymeric molecules [3,6]. Based on their structure, phenolic compounds exhibit different functions, such mechanical support, signalling molecules, colouring agents, or protection against biotic or abiotic stresses (e.g., UV light, pathogens and herbivores, etc.) [2,3,8,9].

Due to the high polyphenol content, fruits and vegetables are recommended for consumption as part of a daily diet [10]. It has been shown, based on epidemiological studies, that a diet enriched in phenolic compounds promotes various health benefits, related to a lower incidence of cardiovascular diseases, cancer, obesity, diabetes, etc. [2,3,11,12]. The health-promoting benefits of phenolic compounds are considered to be a consequence of their antioxidant potential [2,11,13]. These compounds have the chemical ability to react with free radicals produced by oxidative stress, by transferring a hydrogen atom and/or an electron or by chelating some metals, so that the oxidative chain reaction is interrupted [11,14]. Wild fruit species are valuable sources of bioactive compounds with antioxidant potential, among other beneficial and healthy properties [15]. Spontaneous flora includes plants that appear and develop naturally in a specific environment without human intervention [16]. Hawthorn (*Crataegus monogyna*), cornelian cherry (*Cornus mas*), rosehip (*Rosa canina*), and blueberry (*Vaccinium myrtillus*) are fruits widespread in the Romanian spontaneous flora that are rich in bioactive compounds, including phenolic compounds, which are used in traditional medicine for the treatment of various diseases [17,18,19,20].

The biochemical profile of fruits is largely influenced by genotype, but the climatic, geographical, and soil conditions, including temperature, light, precipitation, altitude, and soil fertility are also a factor [15]. Other factors, like the maturity of the fruit [21,22] and the health condition of the host plant [23] can also affect it.

Due to the important properties presented by phenolic compounds, the interest in their identification and quantification has continuously increased [15,24]. The high-performance liquid chromatography with diode array detection (HPLC-DAD) is a popular technique applied for the analysis of phenolic compounds in plant extracts, with the advantages of being simple, not very expensive, and easy to maintain [1,24].

**Figure 1:**
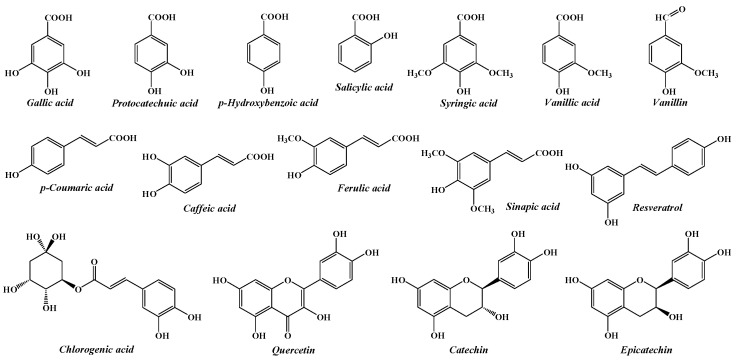
The chemical structures of the phenolic compounds analysed by the proposed HPLC method.

The current study aims to investigate the composition of some native forest fruits, in order to contribute to a better knowledge of the plant resources from the spontaneous flora of Romania, as well as to reveal opportunities for their valorisation. Consequently, a simple and easy-to-implement HPLC method was optimized and validated for the simultaneous determination of 16 phenolic compounds (gallic acid, protocatechuic acid, *p*-hydroxybenzoic acid, vanillic acid, caffeic acid, catechin, chlorogenic acid, vanillin, syringic acid, p-coumaric acid, epicatechin, ferulic acid, sinapic aid, salicylic acid, resveratrol, and quercetin) in four different species of wild fruits from Romania, namely hawthorn (*Crataegus monogyna*), cornelian cherry (*Cornus mas*), rosehip (*Rosa canina*) and blueberry (*Vaccinium myrtillus*). The chemical structures of the investigated phenolic compounds are shown in Figure 1.

These compounds were selected, first of all, to represent key phenolic subclasses, including hydroxybenzoic acids (gallic acid, protocatechuic acid, *p*-hydroxybenzoic acid, vanillic acid, syringic acid, and salicylic acid), hydroxycinnamic acids (caffeic acid, chlorogenic acid, coumaric acid, ferulic acid, and sinapic acid), flavanols (catechin, epicatechin), flavonols (quercetin), stilbenes (resveratrol), and hydroxybenzoic aldehydes (vanillin), and secondly for being commonly found in wild fruits [25,26,27,28,29,30]. Also, the target analytes have been previously documented for their antioxidant, anti-inflammatory, and cardioprotective activities, which correlate with the use of these species in traditional medicine [31,32].

## 2. Results and Discussion

### 2.1. HPLC-DAD Method Optimization

**Figure 2:**
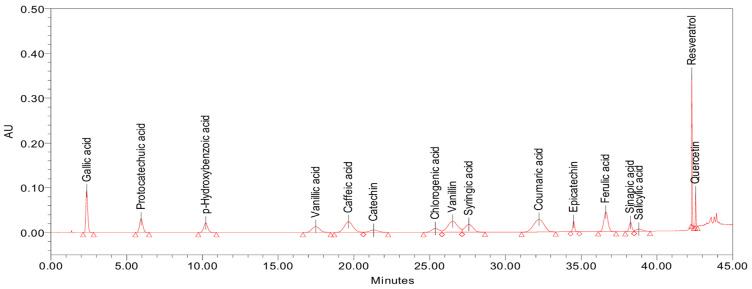
Separation of the 16 phenolic compounds with the optimized gradient (the chromatogram was obtained by injecting 20 µL of an external standard mixture, with each analyte at a concentration of 10 µg/mL and detected at 280 nm).

The starting point for the development of the method was the chromatographic conditions reported by Ghinea et al. [33]. For the separation of the peaks corresponding to the 16 analytes of interest, a gradual change of the elution gradient was performed. An adequate separation, as can be observed in Figure 2, was obtained by applying the step-gradient presented in Table 1.

### 2.2. Identification of the Phenolic Compounds

For the identification of the phenolic compounds, individual standard solutions of the 16 target compounds were injected into the HPLC system. Each phenolic compound was identified by its retention time, presented in Table 2.

### 2.3. Method Validation

#### 2.3.1. Specificity

**Figure 3:**
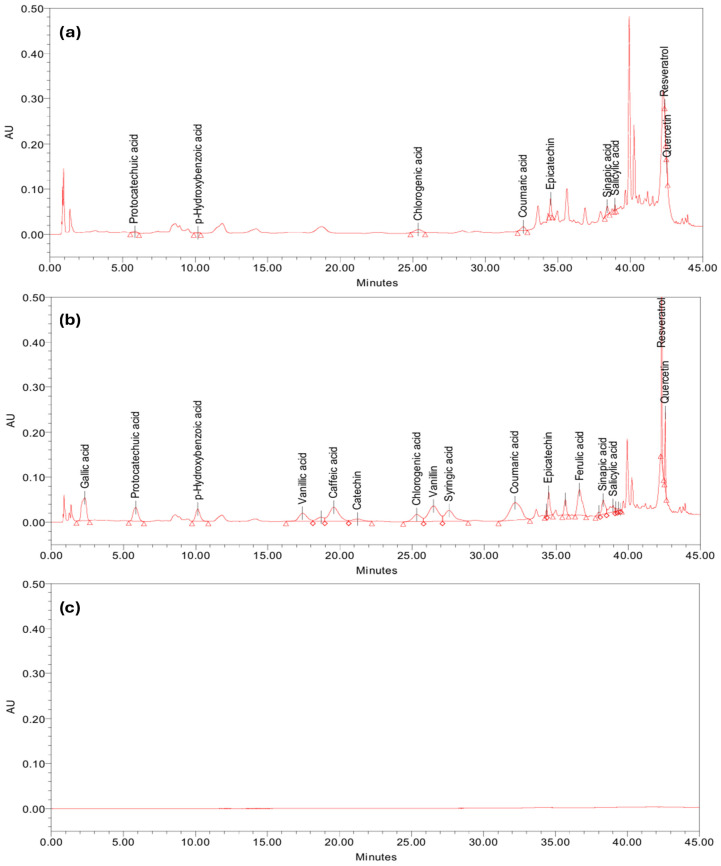
Chromatographic profiles obtained in the specificity test: (**a**) hawthorn sample; (**b**) enriched hawthorn sample; and (**c**) blank (extraction solvent).

The specificity of the analytical method was evaluated by investigating the interferences between the peaks of the compounds of interest with the peaks from the sample matrix or blank. Therefore, the hawthorn extract (test solution) was enriched (spiked) with a concentration of 15 µg/mL of each of the 16 studied analytes. As can be observed in Figure 3, the peaks due to target analytes do not interfere with each other nor with other matrix constituents, thus the method can be considered specific for hawthorn fruit extract and similar matrices. Additional validation would be necessary to confirm the specificity in other types of samples.

#### 2.3.2. Linearity

In order to evaluate the linearity of the relationship between analyte concentration and detector response (peak area), the eight working mixed standard solutions were injected and eluted in the HPLC system, under previously optimized chromatographic conditions. The preparation and composition of the working mixed standard solutions are detailed in Section 3.4. After recording the chromatograms, the calibration curves for each individual analyte were obtained by plotting the peak area as a function of analyte concentration. The least squares method was used to obtain the linear regression equation and the determination coefficient (R^2^), presented in Table 2. The determination coefficients were used to assess the linearity of the detector response. As can be observed in Table 2, very good values of this coefficient were obtained: greater than 0.99 for all analytes, except for quercetin for which a value of 0.98 was obtained. In addition, the quality of fit was assessed by the standard error of estimate (S_y/x_), which represents the residual variability of the observed data from the fitted regression line. The standard error of estimate (S_y/x_) was calculated using the following equation:

$$S_{y/x}=\sqrt{\frac{\sum {\left(y_{o}-y_{p}\right)}^{2}}{n-2}}$$

where *y_o_* represents the observed peak area, *y_p_* is predicted peak area from the regression model, and *n* is the number of calibration points. To compare this error across different compounds, S_y/x_ was divided by the average peak area (average detector response across all calibration points) of each compound and multiplied by 100 to express it as a percentage [34,35]. This shows the relative size of the error compared to the signal. The relative standard error of estimate (S_y/x_) values for all analytes ranged from 1.07 to 5.04% (Table 2), which are within acceptable limits considering the multicomponent nature of the assay. Considering the high determination coefficients (R^2^) and the relative standard error of estimate values, the calibration curves showed a good fit and an acceptable linearity within the studied concentration range.

Peak symmetry (also referred to as the tailing factor) was calculated for all analytes, according to the formula:

$$T=\;\frac{W_{0.05}}{2\times f}$$

where *W*_0.05_ is the width of the peak at 5% of the peak height, and *f* is the distance from the peak maximum to the leading edge at 5% of the peak height. A tailing factor value of 1.0 indicates a perfectly symmetrical peak. As observed in Table 2, symmetry values ranged from 0.90 to 1.32, indicating generally sharp and symmetrical peaks. These are well within the acceptable limits specified by *The International Pharmacopoeia*, which recommends symmetry factors between 0.8 and 1.8 for peaks used in quantitation [36].

Resolution (Rs) between adjacent peaks was calculated manually using peak retention times and baseline widths provided by the chromatography software Waters Empower^®^ 3, according to the following equation:

$$Rs=\;\frac{2\times \left(t_{R2}-t_{R1}\right)}{\left(w_{2}+w_{2}\right)}$$

where *t_R_*_1_ and *t_R_*_2_ are the retention times of two consecutive peaks, and *w*_1_ and *w*_2_ are their respective baseline peak widths (in minutes) as reported by the software. Each resolution value was calculated with respect to the next eluting peak. The chromatographic resolution (Rs) between adjacent phenolic compounds ranged from 0.68 to 5.81 (Table 2). The method achieved an acceptable separation for the majority of analytes, with Rs values exceeding the commonly accepted minimum of 1.5 for baseline separation in most cases. However, some adjacent compounds exhibited lower resolution values: chlorogenic acid–vanillin (Rs = 0.89) and caffeic acid–catechin (Rs = 0.93), with the lowest separation occurred between sinapic acid and salicylic acid (Rs = 0.68). These partially overlapping peaks are likely due to structural similarity and close retention behaviour. Given that this method was developed for the simultaneous profiling of multiple phenolics in complex vegetal samples, rather than for pharmaceutical quantitation, some degree of coelution is acceptable. Similar partial coelution of phenolic compounds has been reported in several studies analysing complex plant extracts by HPLC-DAD [37,38] or waste water [39]. Furthermore, method conditions were optimized to balance separation efficiency with analysis time, which is important for phytochemical profiling studies. For applications where complete baseline separation is essential (e.g., pharmaceutical studies), additional optimization may be needed.

Limit of detection (LOD) values for the 16 phenolic compounds were determined based on baseline noise measurements from six replicate injections of blank (extraction solvent). For each compound, a flat region of the baseline corresponding to the retention time window of the analyte was selected. Peak-to-peak noise was measured manually within this region using the chromatographic software Waters Empower^®^ 3. The standard deviation of the noise (*σ*) was estimated as one-sixth of the peak-to-peak amplitude [40]. The LOD was then calculated using the ICH guideline [41] formula:

$$LOD=\;\frac{3.3\times \sigma }{slope}$$

where the slope was obtained from the calibration curve of each analyte. The resulting LODs ranged from 2.40 to 13.33 ng/mL (Table 2), indicating a high sensitivity of the developed HPLC-DAD method for the quantification of phenolic compounds at a low concentration.

The stability of the working standard solution was evaluated by storing a mixed standard solution containing 16 phenolic compounds at a concentration of 10 µg/mL in the HPLC autosampler maintained at 5 °C. Aliquots of the solution were injected at 0, 6, 12, and 24 h. The peak areas at each time point were compared to those obtained at time zero and the stability was expressed as the percentage difference relative to the initial response. Peak area variations for all 16 analytes remained within ±5% up to 12 h. However, at 24 h, caffeic acid, catechin, epicatechin, and quercetin exhibited peak area variations exceeding ±5%. Therefore, the standard solution is considered stable for up to 12 h under the autosampler conditions.

#### 2.3.3. Precision

The instrumental precision of the analytical method was demonstrated by repeatability (intra-day precision) and intermediate precision (inter-day precision) and was evaluated by relative standard deviation (% RSD) calculated for the peak area of each analyte.

Repeatability was assessed by six replicate injections of a mixed working standard solution at 10 µg/mL on the same day. This approach complies with the ICH requirement of a minimum of six determinations for the same concentration. Additionally, three independently prepared hawthorn extract samples were analysed under the same conditions on the same day to evaluate precision in the matrix.

Intermediate precision was evaluated by analysing the same two mixed standard solutions (1 µg/mL and 10 µg/mL) on three different days. Similarly, the hawthorn extract was freshly prepared and injected on each of the three days to assess day-to-day variability. All analyses were performed under the same experimental conditions (instrumentation, analyst, and chromatographic settings). The obtained data for evaluating the precision of the method is summarized in Table 3. In the absence of fixed numerical limits in ICH Q2(R2) [41], acceptance criteria for precision were set to % RSD values ≤5%, in accordance with the complexity of the plant matrix and the multicomponent nature of the assay. As it can be observed, the % RSD calculated did not exceed 3.15% and, consequently, the analytical method met the precision criteria.

#### 2.3.4. Accuracy

The accuracy was assessed by the degree of recovery of the amounts of analyte added to a test solution. The investigation was carried out at two concentration levels (5 and 15 µg/mL) by enriching a test solution of hawthorn extract with mixed standard solutions. Three samples were prepared for each level of enrichment. The recovery was calculated using the following equation:

% Recovery = (Amount found − Original amount)/Spiked amount × 100

The recovery rates of all analytes, for each level studied, along with their mean value, are presented in Table 4. As can be observed, the recovery rates were generally higher than 90%, which fits the range of 80–120% recommended by to ICH Q2(R1) guideline. But this guideline was developed for pharmaceutical products [41]. Some exceptions were obtained for catechin, ferulic acid, salicylic acid, and quercetin. However, due to the complexity of plant-derived matrices, slightly broader recovery ranges (e.g., 70–120%) are commonly accepted in method validation protocols [42]. This is particularly relevant when analysing low concentrations of multiple analytes in a single run. Similar criteria have been applied in the validation of chromatographic methods for food and feed matrices, where recovery values between 60–140% have been considered acceptable depending on matrix complexity and compound sensitivity [43].

#### 2.3.5. Robustness

The robustness of the method was evaluated by introducing small, deliberate variations in two parameters: injection volume and column temperature. The injection volume was varied by ±2 µL (from the nominal value of 20 µL), and the column temperature was varied by ±2 °C (from the set temperature of 30 °C). These changes were assessed for their impact on the retention time and peak area of all 16 phenolic compounds. Each condition was tested in triplicate. The obtained data are presented in Table 5.

Regarding the effect of column temperature variation, it was observed that retention times showed a varying sensitivity to column temperature, particularly among early-eluting compounds. More precisely, at 28 °C, several analytes showed retention time shifts exceeding 5%, with catechin showing the largest deviation (+9.5%), while at 32 °C, negative retention time shifts were observed (e.g., caffeic acid: −7.19%, and gallic acid: −8.45%), as expected due to increased elution speed. Consequently, strict temperature control is recommended to maintain retention time reproducibility, especially for early-eluting compounds. Despite these shifts, peak area %RSD remained below 2% in all cases, indicating stable detector performance.

In relation to injection volume variation, the peak areas responded predictably, with area ratios for 18 µL and 22 µL injections close to the expected values, ranging from 0.97 to 1.02, indicating excellent proportionality for quantification. Also, all compounds obtained a %RSD of peak area <2%, confirming precise injections at both altered volumes. Thus, the method is robust to small variations in injection volume, with the quantitative response remaining proportional and precise across all analytes.

### 2.4. Application of the Method to Real Samples

The validated analytical method was then applied to the identification and quantification of phenolic compounds from hawthorn (*Crataegus monogyna*), cornelian cherry (*Cornus mas*), rose hip (*Rosa canina*), and blueberry (*Vaccinium myrtillus*) fruit extracts, and the obtained results, expressed in µg/g of fresh weight (FW) ± standard deviation, are summarized in Table 6.

Nine phenolic compounds have been identified for hawthorn, namely: protocatechuic acid, *p*-hydroxybenzoic acid, chlorogenic acid, coumaric acid, epicatechin, sinapic acid, salicylic acid, resveratrol and quercetin. Among the identified phenolics, epicatechin was found in the highest concentration (37.55 µg/g FW), followed by chlorogenic acid (20.82 µg/g FW) and sinapic acid (20.00 µg/g FW). A literature review published in 2011 by Yang and Liu revealed that epicatechin is the main procyanidin found in many *Crataegus* species. Also, chlorogenic acid was frequently identified in studies on the phenolic profile of different parts of *Crataegus* species [44]. Alirezalu et al. [17] studied fruits from 15 different species of hawthorn, including *Crataegus monogyna*, and chlorogenic acid and quercetin were found in all analysed samples.

Concerning the *Rosa canina* extract, 7 phenolic compounds were identified, namely protocatechuic acid, vanillic acid, catechin, vanillin, salicylic acid, resveratrol, and quercetin. Protocatechuic acid, vanillic acid and salicylic acid were also identified by Nowak (2006) in *Rosa Canina* fruit collected from Poland [45]. The highest amount was observed for catechin, with a value of 177.24 µg/g FW. This result is consistent with the findings of previous reported studies conducted on different species of the genus *Rosa* from Lithuania, which revealed catechin as the predominant phenolic compound in all analysed samples [46,47]. Similar results were obtained by Demir et al. [48] for *Rosa canina* fruit from Turkey. Moreover, Elmastaș et al. [49] found catechin as the major flavonoid present at all harvest times of the fruits of different *Rosa* species, including *Rosa canina*. Using an HPLC-LC/MS technique, Stănilă et al. [50] identified the presence of catechin in *Rosa canina* fruit from Romania, also. The current study revealed, as well, the presence of quercetin. Shameh et al. found this flavonoid only in certain investigated rosehip samples, only from certain areas of Iran [51]. The current method allowed the identification and quantification of resveratrol in rosehip fruit. Stănilă et al. [50] reported for the first time in 2015, the presence of resveratrol in this type of plant samples. To the best of our knowledge, the presence of vanillin in *Rosa canina* was not previously reported.

Cornelian cherry (*Cornus mas*) fruit are known to be abundant in phenolic compounds (flavonoids, phenolic acids) [52,53]. The application of the validated method allowed the identification and quantification of gallic acid, *p*-hydroxybenzoic acid, vanillic acid, catechin, chlorogenic acid, syringic acid, epicatechin, ferulic acids, salicylic acid and resveratrol, in the methanolic extract of *Cornus mas* fruit. The presence of this phenolics in cornelian cherry fruit was also confirmed by other previously reported studies [18,54,55]. In the current study, epicatechin showed the highest content (186.0 µg/g FW), followed by the ferulic acid (64.3 µg/g FW) and chlorogenic acid (58.97 µg/g FW).

*Vaccinium myrtillus* fruit were also analysed using the validated method and ten of the target compounds were identified and quantified, more precisely, gallic acid, protocatechuic acid, chlorogenic acid, coumaric acid, epicatechin, ferulic acid, sinapic acid, salicylic acid, resveratrol and quercetin (Table 6). Epicatechin, chlorogenic acid, coumaric acid, ferulic acid, and gallic acid were also identified by Levaj et al. [10] in wild *Vaccinium myrtillus* fruit from Croatia, by an HPLC-DAD method. In addition, these authors found *p*-hydroxybenzoic acid, caffeic acid and catechin, which were not identified in the Romanian wild blueberries analysed in the current study. Similarly, by an HPLC-DAD method, a group of researchers [56] analysed wild blueberries from different regions of Turkey. Gallic acid, chlorogenic acid, coumaric acid, ferulic acid, epicatechin were among the phenolic compounds identified, and small amounts of resveratrol and quercetin were also revealed. Also, the study demonstrated significant differences in the content of phenolic compounds between harvesting regions. *Vaccinium myrtillus* fruit collected from the same area of Romania as in the current study were also investigated by Solcan et al. [19], trough LC–MS analysis. Chlorogenic acid was observed to be the most abundant phenolic compound, and epicatechin, coumaric acid, gallic acid, and protocatechuic acid were also detected. In addition, several flavanols and flavonols which were not the subject of the current investigation, were also detected.

In general, the results obtained are in agreement with other previous reports, regarding the phenolic compounds identified in the plant samples analysed. However, the quantification results are difficult to compare, because different extraction methods and different analysis techniques were used. In addition, variations determined by location, weather, cultivation conditions, maturity stage, environmental stress, etc., significantly influence the concentration of the analysed compounds [55].

## 3. Materials and Methods

### 3.1. Chemicals and Reagents

Standards of phenolic compounds, namely: gallic acid, protocatechuic acid, *p*-hydroxybenzoic acid, vanillic acid, caffeic acid, (+)-catechin, chlorogenic acid, vanillin, syringic acid, coumaric acid, (−)-epicatechin, ferulic acid, salicylic acid, sinapic acid, resveratrol, and quercetin were >95% purity and were purchased from Sigma-Aldrich, (Taufkirchen, Germany). Acetonitrile (HPLC grade), methanol (HPLC grade) and trifluoracetic acid reagent (TFA) were purchased from Merck (Darmstadt, Germany). Ultrapure water with a resistivity of ≥18.2 MΩ·cm at 25 °C and total organic carbon (TOC) ≤10 ppb was prepared with Merck Millipore Simplicity^®^ Water Purification System (Molsheim, France) and was used for solutions preparation.

### 3.2. Berries Samples

For the experiment, four types of forest fruits were used, collected from the spontaneous flora of north-eastern Romania. More precisely, *Vaccinium myrtillus* fruit were harvested from the area 47.52° N, 25.46° E, while *Rosa canina*, *Crataegus monogyna* and *Cornus mas* fruit were harvested from the area 46.09° N, 27.94° E. Fully mature fruits were collected only from visibly healthy plants-showing robust growth, intact foliage and no obvious symptoms of stress. The collected fruits were rinsed with distilled water and dried with a paper towel, then stored in the freezer at −20 °C, until analysis.

### 3.3. Samples Preparation

A solution of MeOH/H_2_O/HCl 1M = 50/42/8 (*v/v/v*) was used as a solvent for the extraction of the phenolic compounds. The frozen fruit material was first crushed and then homogenized using a mortar and pestle until a uniform paste was obtained. A total of 5 g of homogenized plant material was accurately weighed into a 50 mL standard Erlenmeyer flask (conical shape, flat-bottomed). Each sample was mixed with 15 mL extraction solvent (final volume not adjusted), and the flasks were sealed with Parafilm^®^. The resulting mixture was magnetically stirred at 6.85× *g*, using a 2.5 cm Teflon-coated magnetic stirring bar and maintained at a temperature of 50 °C in a water bath for 30 min. After centrifugation at 2236× *g*, 4 °C, for 30 min (Hettich Micro 22 R Centrifuge, Andreas Hettich GmbH & Co. KG, Tuttlingen, Germany), the supernatant was collected, filtered through 0.45 µm PES syringe filter and injected into the HPLC system.

### 3.4. Preparation of Standard Solutions

Individual standard stock solutions in methanol were prepared for each of the 16 studied analytes, at a concentration of 2 mg/mL. Starting with this stock solution and by dilution with a solution of 0.1% (*v*/*v*) TFA in water (the mobile phase A), eight working mixed standard solutions (containing the 16 phenolics) with concentrations of 0.1, 0.5, 1.0, 5.0, 10.0, 25.0, 40.0, and 50.0 µg/mL were obtained. The resulting working standard solutions were filtered through 0.45 µm PES filters before injection into the HPLC system.

### 3.5. Chromatographic Conditions

The analysis of phenolic compounds was realized with the Waters 2695e Alliance HPLC system, coupled with the 2998 photodiode array detector (DAD), controlled by Empower^®^ 3 software (Waters, Milford, MA, USA). The elution was performed using a solution of 0.1% (*v*/*v*) TFA in water as mobile phase A and a solution of 0.1% (*v*/*v*) TFA in acetonitrile as mobile phase B, as previously reported by Ghinea et al. [33]. The separation of the analytes of interest was achieved by the optimization of the elution gradient. As a stationary phase, a capillary C-18 column Waters XBridge (50 × 4.6 mm, 3.5 µm) (Waters, Milford, MA, USA), thermostatically kept at 30 °C, was used. The injection volume was 20 µL.

### 3.6. Validation Methodology

The validation of the optimized method for the simultaneous determination of the 16 phenolics of interest was carried out by taking into account the recommendations of the scientific guideline ICH Q2(R2) of the International Conference on Harmonization (ICH) [34], regarding specificity, linearity, precision (inter-day and intra-day precision), repeatability, and accuracy.

### 3.7. Statistical Data Analysis

Microsoft 365 Excel software was used for data processing (means, standard deviations).

## 4. Conclusions

In the current study, an HPLC-DAD method was optimized and validated for the simultaneous determination of 16 bioactive phenolic compounds in fruits from different spontaneous horticultural species. The method uses a binary gradient system composed of ultrapure water and acetonitrile; both are acidified with trifluoroacetic acid. The separation of the components of interest was carried out on a C18 reverse-phase column, after an elution of 55 min. Following the validation methodology, the proposed method proved to be specific for hawthorn fruit and similar matrices, as well as linear in the studied concentration range, and precise and accurate. Following that, the method was used to assess the phenolic composition of the fruits of *Vaccinium myrtillus*, *Rosa canina*, *Crataegus monogyna*, and *Cornus mas*. Based on the results, epicatechin was found as the main phenolic compound in the fruits of *Vaccinium myrtillus*, *Crataegus monogyna*, and *Cornus mas*, while catechin was the prevailing phenolic compound in the fruit of *Rosa canina*. Cumulatively, the fruit of the species *Vaccinium myrtillus* showed the highest content of phenolic compounds, compared to the other analysed edible fruits. This method will be further extended for the determination of phenolic compounds from other matrices of plant origin.

## Figures and Tables

**Table 1:** The optimized step-gradient program.

Time (min)	Flow Rate (mL/min)	Mobile Phase A (% *v*/*v*)	Mobile Phase B (% *v*/*v*)
0–4	0.7	100	0
5–20	0.7	98	2
27–30	0.7	96	4
32–35	0.7	90	10
40	0.7	80	20
42–45	0.7	0	100
50	0.7	100	0
55	0.7	100	0

Note: % *v*/*v* = volumetric percentage.

**Table 2:** Chromatographic performance and validation metrics for 16 phenolics in HPLC analysis.

No.	Phenolic Compound	RRT	Integration Interval (min)	Peak Symmetry	Rs	Linear Range(µg/mL)	Regression Equation	R^2^	S_y/x_ (Relative %)	LOD (ng/mL)
1	Gallic acid	0.23	2.10–2.80	1.23	4.48	0.1–50	y = 80,163x + 436.49	0.9994	3.04	2.40
2	Protocatechuic acid	0.58	5.50–6.50	1.11	4.03	0.1–50	y = 45,537x + 5907.7	0.9992	3.67	4.24
3	*p*-Hydroxybenzoic acid	1.0	9.70–10.70	1.10	4.78	0.1–50	y = 39,603x + 2826	0.9994	3.25	4.79
4	Vanillic acid	1.73	16.60–18.30	1.15	1.15	0.1–50	y = 45,940x + 4383.4	0.9992	3.64	4.23
5	Caffeic acid	1.91	18.80–20.70	1.18	0.93	0.1–50	y = 98,846x + 6245.2	0.9993	3.47	3.55
6	Catechin	2.07	20.7–22.10	1.02	2.85	0.1–50	y = 20,500x + 2626.2	0.9984	5.04	9.37
7	Chlorogenic acid	2.45	24.60–25.80	1.14	0.89	0.1–50	y = 29,652x + 802.37	0.9999	1.07	2.69
8	Vanillin	2.58	25.80–27.2	1.13	0.76	0.1–50	y = 115,086x + 3665	0.9995	2.81	2.59
9	Syringic acid	2.68	27.20–28.5	0.90	2.45	0.1–50	y = 67,534x − 2633.5	0.9999	1.38	4.42
10	Coumaric acid	3.16	31.20–33.20	1.11	1.60	0.1–50	y = 140,187x + 1580.4	0.999	4.01	2.90
11	Epicatechin	3.36	34.3–34.90	1.14	2.40	0.1–50	y = 19,505x + 775.66	0.9997	2.35	4.62
12	Ferulic acid	3.56	36.10–37.20	1.20	1.83	0.1–50	y = 86,056x + 5954.9	0.9993	3.43	3.13
13	Sinapic acid	3.72	37.90–38.50	1.27	0.68	0.1–40	y = 28,325x − 28.573	0.9994	4.59	5.69
14	Salicylic acid	3.77	38.50–39.50	1.01	5.81	0.5–50	y = 15,111x + 1571.6	0.995	4.50	13,33
15	Resveratrol	4.11	42.20–42.40	1.30	1.53	0.1–50	y = 106,007x + 10,128	0.9992	3.53	2.57
16	Quercetin	4.14	42.40–42.70	1.32	-	0.1–40	y = 39,414x − 59,093	0.9818	4.35	7.80

Note: In all equations, y represents the peak area (in µAU = µV·s), and x is the analyte concentration (in µg/mL). Sy/x = relative standard error of estimate. RTT = Relative retention time calculated with reference to *p*-hydroxybenzoic acid (retention time = 10.279 min). Rs = Resolution values are determined between each peak and the next eluting peak.

**Table 3:** Results obtained for precision assessment, expressed as % RSD retention time and peak area.

No.	Phenolic Compound	Repeatability (% RSD)		Intermediate Precision (% RSD)		
		10 µg/mL (*n* = 6)	Sample (*n* = 3)	1 µg/mL (*n* = 3)	10 µg/mL (*n* = 3)	Sample (*n* = 3)
1	Gallic acid	1.11	nd	0.89	1.63	nd
2	Protocatechuic acid	1.13	1.40	0.50	0.15	1.54
3	*p*-Hydroxybenzoic acid	1.12	1.95	0.87	0.58	0.85
4	Vanillic acid	0.78	nd	0.72	0.85	nd
5	Caffeic acid	1.59	nd	0.28	0.79	nd
6	Catechin	1.54	nd	1.31	1.48	nd
7	Chlorogenic acid	1.47	0.49	1.65	1.50	1.38
8	Vanillin	1.41	nd	0.37	0.36	nd
9	Syringic acid	1.26	nd	1.20	0.77	nd
10	Coumaric acid	1.09	0.45	0.43	0.36	3.15
11	Epicatechin	2.27	0.96	1.22	1.30	1.50
12	Ferulic acid	0.99	nd	0.69	0.43	nd
13	Sinapic acid	0.95	1.54	0.51	0.87	1.88
14	Salicylic acid	1.63	0.81	0.54	0.49	1.96
15	Resveratrol	1.38	1.49	0.23	0.91	2.33
16	Quercetin	2.26	0.82	1.10	2.51	2.83

nd—not detected.

**Table 4:** The results obtained for accuracy evaluation expressed as % Recovery.

No.	Phenolic Compound	Recovery (%)		
		5 µg/mL	15 µg/mL	Mean Value
1	Gallic acid	103 ± 1.8	99.3 ± 2.2	101.0 ± 2.4
2	Protocatechuic acid	94.9 ± 5.1	97.3 ± 2.0	96.1 ± 1.7
3	*p*-Hydroxybenzoic acid	100.0 ± 1.7	95.0 ± 6.4	97.7 ± 3.8
4	Vanillic acid	96.7 ± 1.9	100.7 ± 2.0	98.7 ± 2.8
5	Caffeic acid	98.1 ± 1.7	98.4 ± 0.58	98.25 ± 0.18
6	Catechin	75.6 ± 6.3	76.8 ± 4.56	76.2 ± 0.88
7	Chlorogenic acid	88.04 ± 22.17	92.4 ± 1.7	90.2 ± 3.1
8	Vanillin	99.8 ± 0.34	99.4 ± 3.1	99.61 ± 0.30
9	Syringic acid	98.1 ± 4.9	108.8 ± 1.6	103.5 ± 7.6
10	Coumaric acid	90.7 ± 0.88	96.1 ± 5.5	93.4 ± 3.8
11	Epicatechin	94.4 ± 9.0	91.2 ± 7.6	92.8 ± 2.3
12	Ferulic acid	77.9 ± 1.1	96.6 ± 1.5	87.2 ± 13
13	Sinapic acid	90.1 ± 18.7	105.8 ± 5.7	97.9 ± 11
14	Salicylic acid	87.7 ± 21	77.4 ± 7.4	82.5 ± 7.3
15	Resveratrol	99.5 ± 3.3	101.3 ± 0.14	100.4 ± 1.3
16	Quercetin	62.0 ± 29	73.3 ± 3.9	67.7 ± 8.0

Note: The results are presented as a mean ± standard deviation.

**Table 5:** Summary data from robustness testing: retention time shifts and peak area variability under temperature and volume changes.

No.	Phenolic Compound	Column Temperature Variation					Injection Volume Variation			
		RT Nominal	RT Shift at 28 °C (%)	%RSD Area at 28 °C	RT Shift at 32 °C (%)	%RSD Area at 32 °C	%RSD Area at 18 µL	Area Ratio (18/20)	%RSD Area at 22 µL	Area Ratio (22/20)
1	*Gallic acid*	2.360	4.12	0.35	−8.45	0.26	0.86	1.01	1.36	0.98
2	*Protocatechuic acid*	5.950	6.61	0.15	−7.34	0.28	1.64	0.99	0.30	0.99
3	*p-Hydroxybenzoic acid*	10.279	5.07	0.09	−5.71	0.29	0.72	1.00	0.13	1.00
4	*Vanillic acid*	17.761	7.55	0.24	−5.91	0.90	0.32	1.00	0.16	1.00
5	*Caffeic acid*	19.629	7.50	0.37	−7.19	0.40	0.51	1.00	0.45	1.00
6	*Catechin*	21.317	9.50	1.01	−8.98	1.61	0.71	1.01	2.29	1.01
7	*Chlorogenic acid*	25.353	5.04	0.69	−5.58	0.37	0.25	1.00	0.50	0.99
8	*Vanillin*	26.485	4.24	0.56	−4.45	0.57	0.41	1.00	0.31	1.00
9	*Syringic acid*	27.570	3.79	0.41	−3.81	0.84	0.44	1.01	0.36	0.97
10	*Coumaric acid*	32.443	3.45	0.09	−4.81	1.04	0.31	1.01	0.25	1.00
11	*Epicatechin*	34.516	0.89	0.52	−0.91	0.48	0.14	1.01	0.27	0.99
12	*Ferulic acid*	36.613	1.32	0.25	−1.37	0.22	0.24	1.00	0.09	1.00
13	*Sinapic acid*	38.224	0.66	0.89	−0.96	0.60	0.93	0.98	0.16	0.99
14	*Salicylic acid*	38.798	1.81	1.21	0.13	0.83	0.91	1.02	0.20	1.02
15	*Resveratrol*	42.297	0.10	0.16	−0.17	0.96	0.51	0.99	0.31	0.98
16	*Quercetin*	42.540	0.02	0.72	−0.05	0.41	0.82	0.92	0.66	0.91

Note: RT = retention time of analytes.

**Table 6:** Results of the analysis of phenolic compounds in fruit samples.

No.	Phenolic Compound	Fruit Sample (µg/g fw)			
		*Crataegus monogyna*	*Cornus mas*	*Rosa canina*	*Vaccinium myrtillus*
1	Gallic acid	nd	15.25 ± 0.29	nd	0.56 ± 0.02
2	Protocatechuic acid	3.44 ± 0.05	nd	1.73 ± 0.03	0.19 ± 0.01
3	*p*-Hydroxybenzoic acid	0.33 ± 0.01	5.73 ± 0.11	nd	nd
4	Vanillic acid	nd	1.20 ± 0.05	76.1 ± 1.6	nd
5	Caffeic acid	nd	nd	nd	nd
6	Catechin	nd	5.95 ± 0.14	177.24 ± 0.43	nd
7	Chlorogenic acid	20.82 ± 0.10	58.97 ± 0.67	nd	438.0 ± 6.7
8	Vanillin	nd	nd	26.64 ± 0.87	nd
9	Syringic acid	nd	51.7 ± 0.73	nd	nd
10	Coumaric acid	3.55 ± 0.02	nd	nd	1.55 ± 0.03
11	Epicatechin	37.55 ± 0.36	186.0 ± 2.2	nd	1228 ± 16
12	Ferulic acid	nd	64.3 ± 0.82	nd	303.3 ± 5.5
13	Sinapic acid	20.00 ± 0.31	nd	nd	194.1 ± 3.7
14	Salicylic acid	9.53 ± 0.08	28.7 ± 0.47	30.4 ± 0.48	722 ± 11
15	Resveratrol	1.74 ± 0.03	3.73 ± 0.06	4.69 ± 0.09	5.55 ± 0.13
16	Quercetin	7.96 ± 0.07	nd	10.50 ± 0.26	6.39 ± 0.15

nd—not detected; the results are presented as a mean ± standard deviation.

## Data Availability

The original contributions presented in this study are included in the article; further inquiries can be directed to the authors.
